# Dynamic environmental interactions shaped by vegetative plant volatiles

**DOI:** 10.1039/d2np00061j

**Published:** 2023-02-02

**Authors:** Rocío Escobar-Bravo, Po-An Lin, Jamie M. Waterman, Matthias Erb

**Affiliations:** a Institute of Plant Sciences, University of Bern Bern Switzerland jamie.waterman@unibe.ch matthias.erb@unibe.ch; b Department of Entomology, National Taiwan University Taipei Taiwan

## Abstract

Covering: up to November 2022

Plants shape terrestrial ecosystems through physical and chemical interactions. Plant-derived volatile organic compounds in particular influence the behavior and performance of other organisms. In this review, we discuss how vegetative plant volatiles derived from leaves, stems and roots are produced and released into the environment, how their production and release is modified by abiotic and biotic factors, and how they influence other organisms. Vegetative plant volatiles are derived from different biosynthesis and degradation pathways and are released *via* distinct routes. Both biosynthesis and release are regulated by other organisms as well as abiotic factors. In turn, vegetative plant volatiles modify the physiology and the behavior of a wide range of organisms, from microbes to mammals. Several concepts and frameworks can help to explain and predict the evolution and ecology of vegetative plant volatile emission patterns of specific pathways: multifunctionality of specialized metabolites, chemical communication displays and the information arms race, and volatile physiochemistry. We discuss how these frameworks can be leveraged to understand the evolution and expression patterns of vegetative plant volatiles. The multifaceted roles of vegetative plant volatiles provide fertile grounds to understand ecosystem dynamics and harness their power for sustainable agriculture.

## Abstract 2

Vegetative plant volatiles influence their environment, and *vice versa*. We review these dynamic interactions and identify frameworks that can help to explain volatile emission patterns and their biological functions.

## Graphical abstract

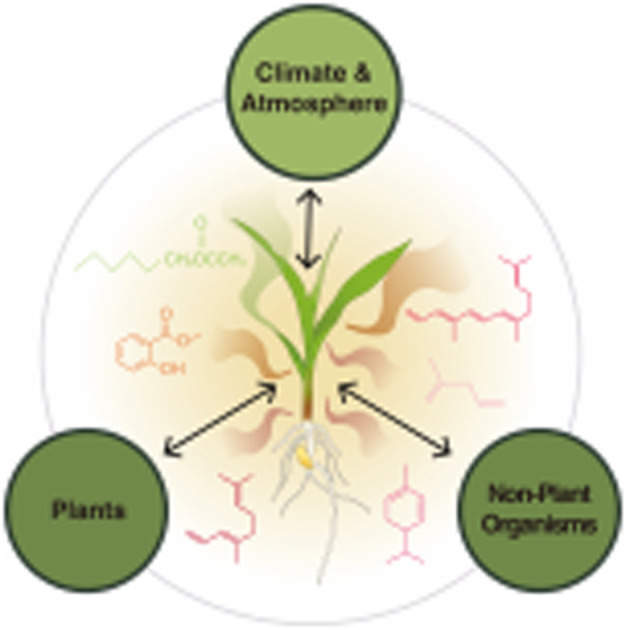

## 1. Introduction

Plants shape terrestrial ecosystems by producing and releasing organic chemicals. Chemicals with low boiling points and high vapor pressure can be released as volatiles^1^ and mediate interactions with the environment at a distance.^2,3^ Floral volatiles are often constitutively released and can serve as attractants of pollinators and defenses against florivores^4^ but can also mediate interactions with other organisms.^5,6^ Vegetative plant volatiles are often highly inducible by biotic and abiotic stressors and play important roles in plant interactions with other organisms and the environment.^2,4,7–9^

Work over the past years has uncovered how vegetative plant volatiles are synthesized and released, how they are modified by their environment, and how they modulate interactions between plants and other organisms.^10–15^ An increasing number of studies have begun to investigate the dynamic patterns that emerge from the multilayered interactions between plants and the biotic and abiotic environment.^16–18^ Understanding the role of vegetative plant volatiles in agroecosystems has become ever more pressing due to climate change, as vegetative volatiles may be used to promote plant resilience in dynamic environments.^19,20^ A robust understanding of the physiological and ecological mechanisms governing volatile-mediated interactions is essential to harness their potential in this context.

Recent reviews have discussed biological functions of vegetative plant volatiles,^21^ the molecular mechanisms of volatile production and emission,^22^ their ecological functions and evolution,^3^ and their physical and chemical behavior in the atmosphere.^23^ Here, we aim to complement this work by reviewing and integrating the most recent findings on the regulation, release, transfer, and biological impacts of vegetative plant volatiles. By highlighting how the environment regulates these volatiles and how they regulate the environment in turn, we aim at gaining a deeper understanding on the dynamics that underpin volatile-mediated ecosystem processes.

## 2. Biosynthesis and release of vegetative plant volatiles

### 2.1 Biosynthesis of vegetative plant volatiles

**Fig. 1:**
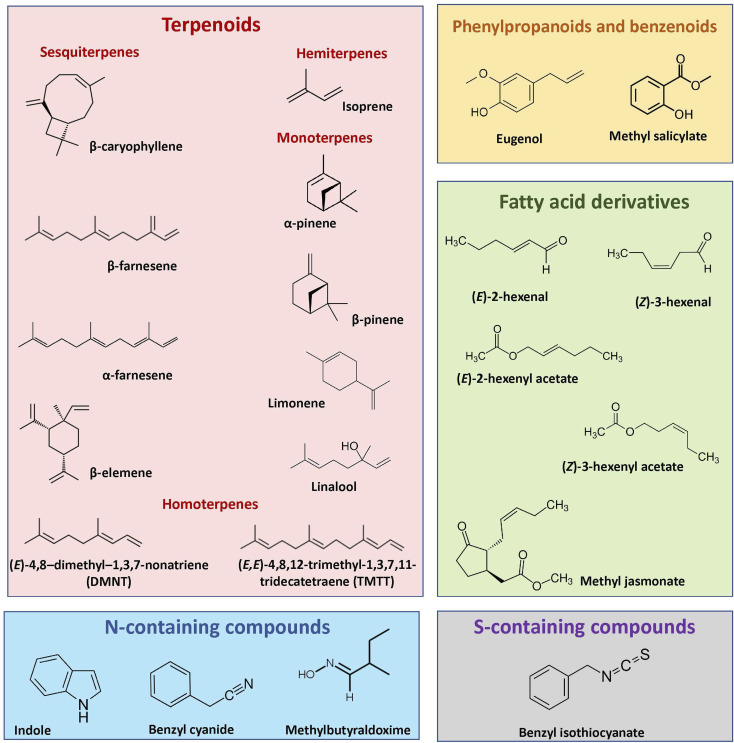
Examples of structures of vegetative plant volatiles that play important roles in plant interactions with other organisms and the environment.

Plants emit diverse blends of vegetative volatiles. These blends include terpenoids, phenylpropanoids/benzenoids, fatty acid derivatives, nitrogen and Sulphur-containing compounds (Fig. 1), all of which are produced *via* distinct biochemical pathways.

Terpenoids comprise over 80 000 known structures.^24^ They are derived from two five-carbon precursors, the isopentenyl diphosphate (IPP) and its allylic isomer dimethylallyl diphosphate (DMAPP), *via* two independent pathways: the mevalonic acid (MVA) and methylerythritol phosphate (MEP). The MVA pathway gives rise to volatile sesquiterpenes, while the MEP pathway provides precursors to volatile hemiterpenes, monoterpenes and diterpenes. In both pathways, IPP and DMAPP are condensed head-to-tail by short-chain prenyltransferases to form the prenyl diphosphate precursors of various chain lengths, including geranyl diphosphate (GPP, C10), farnesyl diphosphate (FPP, C15), and geranylgeranyl diphosphate (GGPP, C20). These precursors are used as substrates by a family of terpene synthases (TPSs) to generate C5-hemiterpenes, C10-monoterpenoids, C15-sesquiterpenoids, or C20-diterpenoids.^25,26^ Many TPSs can synthesize multiple products from a single prenyl diphosphate substrate or accept more than one substrate, thus contributing to terpenoid diversity.^10^ TPS products can be modified further *via* hydroxylation, dehydrogenation, acylation, or other reactions.^11,27^ For instance, oxidative degradation of the TPS2 products (*E*,*E*)-geranyllinalool and (*E*)-nerolidol by two specific P450 monooxygenases results in the biosynthesis of the acyclic homoterpenes (*E*)-3,8-dimethyl-1,4,7-nonatriene (DMNT) and (*E*,*E*)-4,8,12-trimethyltrideca-1,3,7,11-tetraene (TMTT).^11^

Phenylpropanoid and benzenoid compounds are derived from the shikimate pathway. They are defined by their mostly planar and cyclic ring structures with conjugated double bonds and can be classified based on the length of the side chain as phenylpropenes (C6–C3) and benzenoid (C6–C1) compounds.^28^ The first committed step in their biosynthesis is catalyzed by the l-phenylalanine ammonia lyase (PAL) enzyme, which deaminates the aromatic amino acid phenyl alanine to *trans*-cinnamic acid (CA). CA is transformed *via* several enzymatic reactions into *para*-coumaroyl CoA, the general precursor for a wide range of products including anthocyanins, flavonoids, lignin and phenylpropenes. Phenyl propenes are, together with terpenes, major constituents of essential oils, such as methylchavicol or eugenol, which are stored in the trichome glands of basil (*Ocimum basilicum*) leaves.^29,30^ Formation of benzenoids from CA results from the shortening of the propyl side chain by two carbons *via* β-oxidative or non-β-oxidative pathways.^28^

Plants also release several nitrogen-containing volatiles that are derived from amino acid biosynthesis. Indole, a precursor of tryptophan biosynthesis, is produced and emitted by many plant species upon herbivory.^31–33^ Indole synthesis and emission have been best characterized in maize (*Zea mays*), where 1-(2-carboxyphenylamino)-*l*-deoxyribulose-5-phosphate is first converted into indole-3-glycerolphosphate by specific indole-3-glycerolphosphate synthases, and then into volatile indole by the action of an indole-3-glycerolphosphate lyase.^34^ Aldoximes and nitriles occur as intermediates in the biosynthesis of several nitrogenous defense compounds such as cyanogenic glycosides, hydroxynitrile glycosides and camalexin. The biosynthesis of these compounds has been well characterized in Western balsam poplar (*Populus trichocarpa*). Volatile aldoximes such as 2- and 3-methylbutyraldoxime, and nitriles such as benzyl cyanide, which are induced upon herbivory, are derived from amino acids by the action of two cytochrome P450 (CYP) monooxigenases of the CYP79 family.^35^ Nitriles can be produced by two additional P450 enzymes belonging to the CYP71 family, which catalyze the conversion of aldoximes to nitriles.^36^

Volatile fatty acid derivatives include green leaf volatiles (GLVs), consisting of C6 compounds including alcohols, aldehydes, esters, as well as the phytohormone methyl jasmonate (MeJA). GLVs and jasmonates are produced *via* the lipoxygenase (LOX) pathway, which begins when C18-polyunsaturated fatty acids, linoleic and linolenic acids, are cleaved from cell membranes by lipases and dioxygenated by either 9- or 13-LOXs to form 9- and 13-hydroperoxides, respectively. These hydroperoxides act as substrates in the hydroperoxide lyases (HPLs) and allene oxide synthases (AOSs) pathways to produce GLVs and jasmonic acid (JA), respectively. For GLV biosynthesis, 13-hydroperoxy octadecatrienoic acid is cleaved by HPL to form (*Z*)-3-hexenal, which is spontaneously or enzymatically converted into other C6 compounds, including (*E*)-2-hexenal, (*Z*)-3-hexenol and (*Z*)-3-hexenyl acetate.^37^ Biosynthesis of MeJA is synthesized in the peroxisome from a 13-hydroperoxide intermediate *via* sequential reduction and β-oxidation steps, which results in the formation of JA, and it is followed by the methylation of JA by a JA carboxyl methyltransferase.^38^

Volatiles can also result from the breakdown of plant secondary metabolites. Isothiocyanates, for instance, are sulfur-containing volatiles that are characteristic of the Brassicaceae and produced by the myrosinase-mediated hydrolysis of glucosinolates.^39^ Glucosinolates are a structurally diverse group of β-thioglucoside-*N*-hydroxysulfates classified according to their precursor amino acid as aliphatic (from alanine, valine, leucine, isoleucine, or methionine), aromatic (from phenylalanine or tyrosine), or indolic glucosinolate (from tryptophan). Their biosynthesis starts with the modification of the precursor amino acid to form the glucosinolate core. In some instances, side chain elongation or secondary modifications can occur prior to and after core biosynthesis.^40^ Plants producing these compounds also synthesize myrosinases, which are specific β-thioglucosidases that are kept separately from their substrates. When plant tissues are disrupted, the myrosinases hydrolyse the glucose residue of glucosinolates releasing an unstable intermediate, which rearranges to form non-volatile and volatile isothiocyanates and nitriles.^40^

In summary, vegetative plant volatiles are produced through several different pathways and mechanisms. While some volatiles are synthesized from simpler precursors, others are breakdown products of more complex compounds. These diverse origins contribute to the temporal, spatial and environmental complexity of plant volatile blends, and provide many independent attack points for ecological and evolutionary processes to shape these blends.

### 2.2 Mechanisms of vegetative plant volatile release

**Fig. 2:**
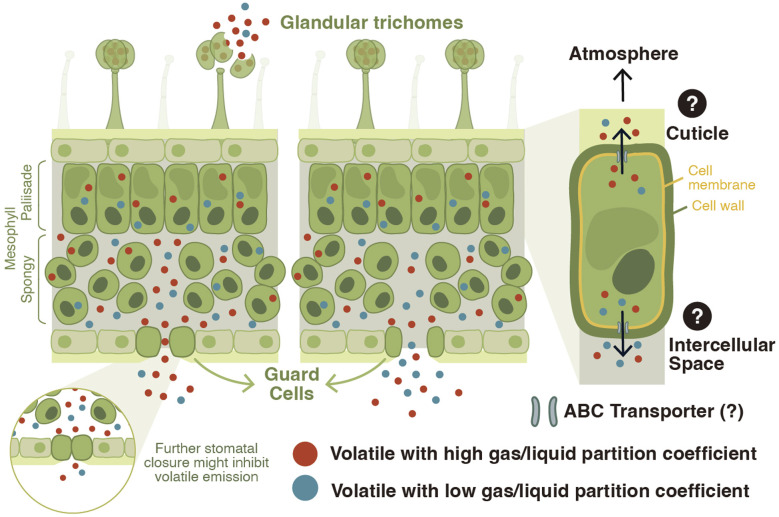
Model of vegetative plant volatile transport and release. Vegetative plant volatiles that are produced by mesophyll cells exit the cells and accumulate in intercellular spaces. The mechanisms of volatile transport from the cytosol across the plasma membrane into the intercellular spaces have not been characterized in the leaves. However, this process might be actively controlled by protein transporters, such as ATP-binding cassette (ABC) transporters, as it is the case in flower tissues. The cuticle might act as an active sink/concentrator for volatiles altering their emission, internal pools, and biosynthesis. Volatiles exit the leaf *via* the stomata, and possibly the cuticle. Volatile release *via* stomata is determined by stomatal aperture and the gas/liquid phase partition coefficient, or Henry's law constant (*H*). Volatiles with a high *H* may reach a higher pressure in the intercellular gas phase and may thus be released even when stomata are partially closed. Volatiles with a low *H* reach lower intracellular pressure and are thus more sensitive to changes in stomatal closure. Glandular trichomes are epidermal structures that can produce and store volatile organic compounds. They release volatiles upon tissue disruption by wounding and/or herbivory.

The mere production of a volatile organic compound is not sufficient for its release into the environment. Vegetative plant volatiles produced in the epidermal cells of roots^25^ or in the mesophyll cells of green-leaf tissues have to cross different cellular boundaries such as the cell membrane and wall, the intercellular spaces, and the stomata or cuticle before being released into the environment (Fig. 2).^41–43^ Depending on their physicochemical characteristics, volatile compounds can accumulate in the lipid or aqueous phase.^44^ The emission of volatiles driven solely by diffusion can lead to toxic volatile accumulation in membranes because of preferential partitioning of some of these compounds into lipid bilayers.^45^ To enhance export and avoid toxicity, volatile phenylpropanoid and benzenoid compounds are transported across the plasma membrane by an adenosine triphosphate–binding cassette (ABC) transporter in *Petunia hybrida* flowers.^12^ Recent discoveries have furthermore shown that a pleiotropic drug resistance transporter 3 (AaPDR3) from the ABC transporter superfamily is involved in the transport of the sesquiterpene β-caryophyllene in annual mugwort (*Artemisia annua*) trichomes.^46^ Thus, volatile transport can be regulated *via* membrane transporters.

Following their transfer across membranes, leaf volatiles have three routes to reach the atmosphere: diffusion across the cuticle, release *via* stomata, or direct release from wounded cells. In *Petunia hybrida* flowers, the cuticle acts as a sink/concentrator for volatiles, and a reduction in cuticle thickness alters volatile emission, internal pools and biosynthesis.^47^ In the leaves, significant correlations have been observed between stomatal aperture and volatile emission.^42,43,48^ When stomata are closed, sesquiterpenes for instance can accumulate in the leaf, suggesting that their release is constrained by stomatal aperture.^42,43^ Detailed kinetic analyses in *Pinus pinea* revealed a partial disconnect between the release of the monoterpene β-ocimene and stomatal conductance.^49^ This phenomenon may be explained by the higher gas/liquid phase partition coefficient of this volatile, also called Henry's law constant (*H*). Volatiles with a high *H* accumulate at higher intercellular partial pressure, resulting in less sensitivity to stomatal closure^49^ (Fig. 2).

Some plant species have specialized secretory structures in their aerial tissues such as resin ducts, idioblasts, and/or glandular trichomes where they produce and store volatiles. Glandular trichomes are hair-like epidermal structures that can produce and accumulate essential oils.^30^ In the tomato clade of the *Solanum* genus, for instance, plants produce leaf glandular trichomes that synthesize and store volatile terpenoids.^50,51^ Emission of type-VI trichome-derived terpenoids is very low in intact tomato leaves. Upon trichome disruption, however, these compounds are strongly emitted, suggesting this as the main mechanism for volatile release^52^ (Fig. 2). In tomato, after biosynthesis, volatile terpenoids are directed into an intercellular storage cavity outside of the gland cells.^53^ It has been suggested that the low emission from intact trichomes might be explained by the cell wall material surrounding this storage cavity, which could prevent the escape of the volatiles and re-entry into the secretory cells.^54^

In summary, plants can release volatiles *via* several different routes, including active transport and passive diffusion (Fig. 2). By consequence, plants can potentially regulate volatile release *via* biosynthesis, transporter activity, stomatal opening, and cuticular composition. The latter processes are intimately linked to other biological functions. Transporters, for instance, are likely to transport other small molecules, stomata are essential for gas exchange, and the cuticle provides and important physical barrier against abiotic and biotic stresses. The regulation of volatile release through processes such as stomatal opening and cuticular structure is thus associated with functional trade-offs that may complicate associated evolutionary trajectories. Therefore, the regulation of volatile release for compounds that can be produced *de novo via* the production of specific enzymes may be accomplished more effectively by regulating their biosynthesis. Storage and wound release are also an efficient way of tailoring volatile release to environmental stress.

## 3. Environmental regulation of vegetative plant volatile biosynthesis and release

### 3.1 Regulation by biotic stressors

#### 3.1.1 Herbivores

**Fig. 3:**
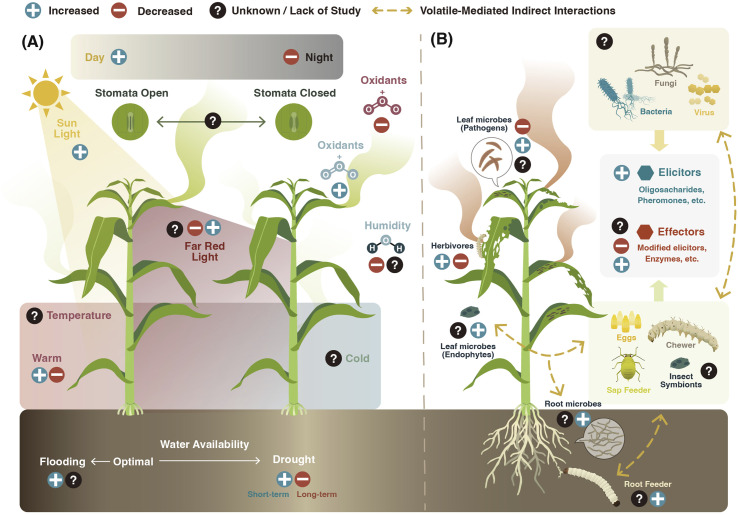
Environmental regulation of vegetative plant volatiles. Induction (+) or suppression (−) of vegetative plant volatile emission can be modulated by (A) abiotic and (B) biotic factors. (A) Light intensity and spectrum, photoperiod, temperature, water availability, air humidity, and oxidants such as ozone (O_3_) can control constitutive and inducible volatile biosynthesis and release. (B) Herbivores and microorganisms can use elicitors and effectors to either induce or suppress the biosynthesis and emission of vegetative plant volatiles. Furthermore, they can wound plants and thereby trigger volatile release. Root and leaf herbivores and microbes can interact *via* the plant and thereby trigger distinct volatile responses.

Vegetative plant volatiles can be emitted constitutively or upon herbivore attack.^3,22^ The production of herbivore-induced volatiles is tightly regulated through plant hormonal signaling mainly controlled by the phytohormones jasmonic acid (JA), salicylic acid (SA), abscisic acid (ABA), and ethylene (ET).^55^ Herbivore-induced plant volatiles can be a highly adaptive plant trait,^56^ playing an important role in the plant defense arsenal against herbivores (see Section 5). Recent studies, however, have demonstrated that herbivorous arthropods can also modulate plant volatile emissions for their own benefit^43,57^ (Fig. 3B).

Volatile composition and emission by plants vary depending on the identity of the attacker. For example, feeding by thrips (cell content-feeder), spider mites (cell content-feeder), or aphids (phloem-feeder) in cucumber (*Cucumis sativus*) leaves induces the expression of different terpene synthases, which translates into different volatile emission patterns.^58^ In addition, attacks by chewing herbivores tend to trigger higher emission of volatiles than by piercing-sucking insects, suggesting that different types and levels of damage induce distinct volatile responses in plants.^8,59^ Simultaneous or sequential attack by multiple herbivores can result in distinct volatile emissions as well.^60,61^ Aboveground attack by the fall armyworm (*Spodoptera frugiperda*), for instance, induces volatile emission in the roots of maize (*Zea mays*) plants that repels Western corn rootworm (*Diabrotica virgifera virgifera*) larvae. Yet, when the Western corn rootworm feeds on the plant first, the induction of the repellent volatiles is suppressed.^61^

The modulation of vegetative plant volatile emission by herbivores is mediated by the recognition of damage-associated molecular patterns as well as herbivore-associated molecular patterns present in the oral secretions, saliva, ovipositional fluids, digestive waste products, and pheromones of the herbivore.^62^ Depending on their ability to induce or suppress plant defense responses, these are called elicitors or effectors.^63^ Herbivore-derived elicitors like β-glucosidases,^64^*N*-acyl-glutamines such as volicitin,^65^ and cyclic disulfide-bridged inceptin-related peptides^66^ found in the oral secretions of lepidopteran larvae, as well as monounsaturated disulfooxy fatty acids (caeliferins) present in the oral secretions of non-lepidopteran insects,^67^ have all been shown to induce volatile emissions in plants. Recently, four oligosaccharides detected in the oral secretions of the cotton leafworm (*Spodoptera littoralis*) larvae induced volatile emissions in Tansy (*Tanacetum vulgare*) plants.^68^ Herbivore-derived proteins from eggs^69,70^ and frass^71^ modulate volatile emissions as well. For instance, an annexin-like protein “diprionin” isolated from egg-associated secretions in sawfly (*Diprion pini*) was recently found to trigger (*E*)-β-farnesene emission in Scots pine (*Pinus sylvestris*).^70^ Interestingly, volatiles released by insects, such as sexual and aggregation pheromones, can also modulate volatile emissions by enhancing herbivory-induced plant defense responses^72^ and volatile release.^73^

Herbivore-derived effectors can suppress volatile emissions.^63^ Silkworms (*Bombix mori*),^74^ the fall armyworm (*S. frugiperda*),^57^ velvet bean caterpillar (*Anticarsia gemmatalis*),^75^ tomato fruitworm (*Helicoverpa zea*), and tobacco hornworm (*Manduca sexta*)^76^ have been all reported to suppress induced volatile emissions in plants. In *B. mori* larval oral secretions, a fatty acid hydroperoxide dehydratase (BmFHD) suppresses GLVs production.^74^ Similar enzymes are present in the oral secretions of other lepidopteran species.^77^ The truncated form of inceptin (Vu-In^−A^) produced by the legume-specialist velvet bean caterpillar antagonizes inceptin-induced responses, including the production of the homoterpene DMNT in cowpea (*Vigna unguiculata*).^75^ Recently, in *H. zea*, the salivary enzyme glucose oxidase has been shown to inhibit volatile emission in different host plants.^43^ This inhibition was linked to the suppression of stomatal aperture.^78^ Besides the induction or suppression of volatile emissions, some herbivores can modulate the chemical profile of the released plant volatile compounds. In *M. sexta*, the larval oral secretions are enriched in an (3*Z*):(2*E*)-hexenal isomerase that modulates the release of plant GLVs by re-arranging (*Z*)-3-hexenal to (*E*)-2-hexenal.^79^

In summary, the release of plant volatiles following herbivore attack is strongly influenced by the action of elicitors and effectors (Fig. 3B). Elicitors act by activating the biosynthesis of plant volatiles, but to what extent these compounds also regulate transport and release processes is not well established. Herbivore effectors are known to target both the biosynthesis and volatile release processes. Understanding the interplay between elicitors and effectors in regulating volatile release is an important research frontier.

#### 3.1.2 Microorganisms

Similar to their response to herbivores, plants can emit specific blends of volatiles when coming into contact with pathogenic or beneficial microbes^80^ (Fig. 3B). Emission of microbe-induced volatiles is regulated by the recognition of microbial-associated molecular patterns and the activation or suppression of plant defense signaling pathways.

Pathogenic bacteria can modulate volatile release in plants. A virulent strain of *Pseudomonas syringae*, for instance, increases the emission of TMTT, β-ionone, and α-farnesene in Arabidopsis.^81^ In tobacco (*Nicotiana tabacum*), both avirulent and virulent *P. syringae* strains induce the emission of MeSA, monoterpenes, and sesquiterpenes in infected plants.^82^ Gram-negative bacterial pathogens such as *P. syringae* have a type III secretion system (a molecular syringe) that injects effectors into the host cell to suppress the plant immune system and promote bacterial infection. Tobacco plants treated with *P. syringae* mutants deficient in this secretion system emit less volatiles upon infection.^82^ More recently, it has been shown that plant recognition of one of these injected *P. syringae* effectors, *AvrRpm1*, triggers the emission of vegetative volatiles in Arabidopsis.^83^

Vegetative plant volatile emissions can be also regulated by fungal infections.^84^ Infection with fungal pathogens elicits greater GLVs emissions than wounding alone or insect herbivore attack.^8^ Although the mechanisms of this phenomenon are still unknown, it has been suggested that enhanced GLVs production might be controlled by the action of fungal effectors. In nature, however, pathogen infections can co-occur with herbivore attacks resulting in distinct volatile profiles.^85^ These patterns may be explained by hormonal cross-talk.^85–87^ For instance, the rust fungus (*Melampsora laricipopulina*) reduces the biosynthesis and emission of herbivore-induced plant volatiles in black poplar (*Poplus nigra*) by activating the SA pathway and suppressing JA associated defenses.^87^

Plant viruses regulate vegetative plant volatile emissions as well.^88^ For instance, the *Tomato spotted wilt virus* (TSWV), mainly transmitted by Western flower thrips (*Frankliniella occidentalis*), reduces the expression of terpene synthases and emission of monoterpenes in thrips-infested pepper (*Capsicum annuum*) plants. This suppression is mediated by a non-structural protein of TSWV that directly interacts with the MYC2 transcription factor, a JA signaling regulator.^89^ MYC2 is also targeted by the viral genetic factor βC1 of *Tomato yellow leaf curl China virus*, which suppresses plant terpene biosynthesis in Arabidopsis and *Nicotiana benthamiana* plants.^90^ In a recent study, infection with the *Cucumber mosaic virus* was found to reduce the production of the monoterpenes 2-carene and β-phellandrene through the action of a viral 2b RNA silencing suppressor protein in tomato (*Solanum lycopersicum*), implicating microRNAs in the regulation of volatile emissions as well.^91^

Apart from pathogens, microbes that live in symbiosis with plants and herbivores can also influence constitutive and stress-induced vegetative plant volatiles. Root colonization by nitrogen-fixing rhizobia, for instance, increases indole and MeSA emission while suppressing β-caryophyllene production in leaves of JA-induced lima bean plants (*Phaseolus Lunatus*).^92^ Soil inoculation with the rhizobacteria *Pseudomonas fluorescens WCS417r* and *P. putida SJ04* enhances aboveground emissions of menthone, menthol, and pulegone terpenoid compounds in peppermint (*Mentha* × *piperita*) plants.^93^ In addition, colonization with rhizobacteria alters the expression of genes responsible for the synthesis of sesquiterpenes and indole in maize roots.^94^ Similar systemic effects have been also observed for aboveground colonizing microbes. The endophytic fungus *Neotyphodium uncinatum*, which colonizes the aerial parts of the grass hybrid *Festuca pratensis* × *Lolium perenne*, can reduce the emission of root volatiles.^95^ Effects of beneficial microbes might be mediated by the increased N availability and other resources needed for volatile biosynthesis,^96^ but also by the modulation of defense signaling.^97^ In the case of insect symbionts, the presence of the endosymbiont *Hamiltonella defensa* in pea aphid (*Acyrthosiphon pisum*) has been shown to compromise plant indirect defenses (*i.e.*, parasitoid attraction) by altering volatile emission upon aphid feeding.^98^

In summary, plant pathogenic and beneficial microbes can induce and suppress plant vegetative volatiles, locally or systematically, *via* microbial elicitors and effectors that target plant defense signaling. Recent studies have shown that microbes present in herbivores and parasites of herbivores can also affect the volatile emissions of their host plant, thus adding an additional layer to the biological regulation of plant volatile release.^99^

### 3.2 Regulation by abiotic stressors

#### 3.2.1 Temperature

Environmental temperature is an important determinant of vegetative plant volatile emissions (Fig. 3A). Long-term increased temperature, for instance, enhances the emission of plant-derived terpenoids and GLVs in subarctic ecosystems.^16,100^ The effects were more pronounced in the presence of herbivores.^16^ Synergism between warming and herbivory was also observed in dwarf birch (*Betula nana*).^17^ These synergistic interactions might be explained by temperature effects on plant defense signaling, volatile biosynthesis, volatilization and diffusion, herbivore physiology, oil organic matter mineralization rates, and increased nutrient availability.^19,101^ In *Empetrum hermaphroditum*, *Cassiope tetragona*, and *B. nana*, warming treatment did not affect volatile emissions directly, but it was affected by increased nutrient availability.^102^

Short periods of high temperature can affect plant volatile emissions. In the Mediterranean shrub (*Halimium halimifolium*), a 10 days heat wave resulted in an increase in volatile emission during the first two days.^103^ A ^13^C-labeling approach revealed that plants allocated more carbon into *de novo* biosynthesis of plant volatiles. Carbon investment for *de novo* volatile biosynthesis was also enhanced in *Salix* and *Betula* spp upon warming, suggesting the role of these volatiles in stress protection.^104^ Overall, plant volatile emissions increase at higher temperatures despite a negative carbon balance under heat stress. The effect of temperature on plant volatile emission is likely associated with the level of temperature changes and the physiological tolerance of each plant species.

Short-term heat stress can modulate vegetative plant volatile emissions *via* multiple pathways. Heat stress triggers emission of oxygenated volatiles, such as acetaldehyde. The enhanced acetaldehyde emission can be explained by induced ROS accumulation and fatty acid peroxidation.^105,106^ Heat stress also enhances the emission of methanol in some plant species.^103^ Methanol can originate from the action of methyltransferase proteins and protein repair reactions, both induced under heat stress.^107^ Emission of terpenoids can be also modulated by the direct heat effects on the volatility of these compounds, and indirectly through the induction of *de novo* biosynthesis.^44^ Nevetheless, work is needed to understand how temperature influences the biosynthesis, transport, and release of vegetative plant volatiles.

#### 3.2.2 Water availability

Water availability can modulate plant volatile emissions (Fig. 3A). Drought, in particular, regulates plant volatile production and release. In white spruce (*Picea glauca*), water deficit resulted in 70% photosynthesis reduction, while only decreasing 37% of the metabolic flux through the MEP pathway that governs isoprenoid biosynthesis.^108^ In Scots pine seedlings, water deficit decreased the emission of some sesquiterpenes, but not isoprene, monoterpenes, and oxygenated compounds.^109^

In addition to soil water availability, air humidity can also influence plant physiological processes itself. For example, high humidity usually leads to larger stomata and pores, as well as impaired stomatal function.^110^ This can affect photosynthetic processes and volatile biosynthesis,^111^ and also volatile release rate through changes in stomatal aperture.^42^ Low air humidity effects on stomatal closure might lead to lower ozone uptake by plants and, therefore, higher ozone concentration in the atmosphere.^44,112,113^ Increased ozone levels can, in turn, alter plant volatile production and release (see Section 3.2.4).

As drought is often associated with heat stress,^114^ many studies have investigated how the combination of these two stressors influence plant volatile emissions.^2,19^ In piñon pine (*Pinus edulis*), elevated temperature alone increased monoterpene emissions, but the opposite was observed in combination with severe water deficit conditions, when the carbon assimilation rates drop to zero.^115^ In the tropical rainforest tree, *Couepia longipendula*, high leaf temperatures and water deficit resulted in a strong decrease in photosynthesis, transpiration, and emissions of volatile terpenoids with a simultaneous stimulation of GLVs.^116^ Lower terpenoid emissions under severe abiotic stress conditions are likely caused by lower photosynthetic activity, higher membrane peroxidation, lower net carbon assimilation, excessive accumulation of reactive oxygen species, and higher lipid peroxidation.^116^

Drought can also modulate herbivore- and microbial-induced plant volatile emissions by itself.^117^ In tea (*Camellia sinensis* var. *sinensis*) plants, for instance, water deficit enhances MeJA-induced volatile emissions.^118^ In tomato, water deficit can increase the emission of herbivore-induced vegetative plant volatiles that function as repellents to insects.^119^ By contrast, water deficit reduces the emission of MeSA upon infection with the bacterial pathogen *Candidatus Liberibacter* in citrus plants.^120^ In general, drought can be expected to suppress the emission of leaf volatiles with a low Henry's law constant (*H*) by increasing stomatal resistance (see Section 2.2).

Compared to water deficit, less is known about the effect of water saturation and flooding on vegetative plant volatile emission. Flooding significantly changes the quality of the volatile blend in maize plants and reduces total volatile emission in certain maize genotypes but not others. However, when flooded maize plants are damaged by *S. frugiperda* caterpillars, they produce a significantly higher amount of volatiles.^121^

#### 3.2.3 Light

The light environment can strongly modulate vegetative volatile emission in plants *via* different mechanisms (Fig. 3A). Light controls the metabolic flux from photosynthesis to terpenoid biosynthesis, as the Calvin cycle delivers the starting substrates for the MEP pathway.^111^ Accordingly, the promoters of many TPSs contain multiple motifs related to light responsiveness and circadian rhythmicity.^58^ This can partially explain the diurnal fluctuations in terpene biosynthesis rates that translate into greater emissions during the day than during the night.^122^ Apart from this strong regulatory mechanism over the volatile biosynthetic machinery, light can control the emission of volatiles with a low Henry's law constant (*H*) (see Section 2.2) *via* diurnal changes in stomatal aperture.^42,49^ Increasing light intensity has been shown to augment type-VI glandular trichome density and, concomitantly, the production of trichome-derived volatile terpenoids in tomato plants,^123^ indicating that light can not only control volatile production but their storage structures as well.

Changes in light spectral composition can also modulate vegetative plant volatile emissions. In dense canopies, the absorption of red (R) and reflection of far-red (FR) light by photosynthetic tissues causes a decrease in R to FR ratios. Plants respond to this light shift by investing in growth to outcompete their neighbors^124^ and suppressing defenses.^125^ Both constitutive and inducible volatile emission are decreased in *Arabidopsis thaliana* upon low R : FR ratio treatment.^126^ In tomato, the JA-induced production of volatile compounds is also modulated by low R : FR ratios or genetic inactivation of the FR-associated photoreceptor, resulting in specific volatile blends.^127^ In addition, decreased proportions of ultraviolet radiation A, blue and green-yellow light, together with increases in R and FR, can decrease the concentrations and emissions of the phenylpropanoid eugenol and the monoterpenes linalool and 1,8-cineole in basil (*Ocimum basilicum*).^128^ Enhanced blue light treatment, however, can decrease the emission rates of monoterpenes in Norway spruce (*Picea abies*).^129^ FR supplementation can enhance the emission of maize (*Z. mays*) volatiles in response to herbivory or volatile cues emitted by neighboring plants.^130^ These effects may be in part explained by the positive impact of supplemental FR on stomatal conductance and photosynthetic rate.^130^ Similar effects have also been found in goldenrod (*Solidago altissima*), where FR enhanced plant volatile emissions in response to volatile cues from non-damaged neighbors.^131^

#### 3.2.4 Ozone and other stresses

Tropospheric ozone (O_3_) is a major phytotoxic air pollutant.^132^ Ozone exposure alone has been shown to induce the synthesis and release of vegetative plant volatiles in some cases,^133^ including herbivore-induced plant volatile emissions^134^ (Fig. 3A). In winter cress (*Barbarea vulgaris*), ozone treatment reduced sesquiterpene emissions in plants attacked by diamondback moth (*Plutella xylostella*) caterpillars.^135^ In southern blue gum (*Eucalyptus globulus*), acute ozone treatment combined with leaf wounding enhanced isoprene, GLVs, monoterpenes, and sesquiterpenes emissions.^136^ Exposure to ozone can enhance herbivore-induced plant volatiles leading to reduced herbivory in black mustard (*Brassica nigra*).^134^ Yet, in other scenarios, ozone exposure can reduce volatile biosynthesis and release. The effects have even been shown to vary in the same plant species depending on the season.^137,138^

The impact of other abiotic factors, such as minerals, heavy metals, low temperature, rain, and wind on plant volatile emission is less clear. Salt stress increases the emission of sesquiterpenes and certain monoterpenes in tropical daisies (*Egletes viscosa*).^139^ In addition, plants can respond to cold stress by emitting more volatile terpenoids and GLVs.^140^

In summary, abiotic factors strongly modulate vegetative plant volatile emissions. Given the diverse origins and release pathways, it is not surprising that the changes in plant metabolism and physiology triggered by variations in the abiotic environment leads to distinct volatile release patterns. Further work on the link between the perception of abiotic stress and the regulation of plant volatile release will be an important next step to understand the adaptive context of these responses.

## 4. Transfer, degradation, and uptake of vegetative plant volatiles after release

As vegetative plant volatiles are released into the atmosphere, these compounds must travel through the atmosphere to their targets, such as neighboring plants or sensory organs of other organisms, to realize their biological function.^141,142^ Several environmental factors can modulate this transfer.

### 4.1 Oxidants

Plant volatiles can react with oxidants (*e.g.*, OH, NO_3_, O_3_) in the atmosphere (See ref. 148 for detailed graphics). For example, isoprene has been shown to be reactive with OH^13^ and terpenoids with O_3_.^143,144^ These oxidative processes are responsible for the majority (68–69%) of biogenic secondary aerosols,^145^ which are important for absorbing radiation from the sun, the formation of cloud droplets, precipitation patterns and humidity in general.^146,147^ Vegetative plant volatiles can also contribute to the generation of atmospheric oxidants. The oxidation of plant volatiles can generate peroxyl radicals that react with NO to form NO_2_, which is then photolyzed to generate O_3_.^148^ It is estimated that forest-emitted isoprene contributes to the generation of 15–18% of the tropospheric ozone.^149^

Atmospheric oxidants are also involved in reducing the availability of plant volatiles.^150^ Ozone fumigation reduces the atmospheric concentration of mono- and sesquiterpenes released by oilseed rape (*Brassica napus*). The reduction in sesquiterpene concentration was likely linked to ozonolysis at the leaf surface, whereas reduction in monoterpene concentration was linked to reactions with ozonolysis-derived OH radicals in the air.^151^ It was also shown that increased ambient ozone reduces the adsorption of the monoterpene myrcene in white cabbage (*Brassica oleracea* convar. *capitata* var. *alba*).^152^ The reactivity of vegetative volatiles in the atmosphere is compound-specific, with reaction lifetimes that range from days to seconds.^150^

### 4.2 Air humidity

Air humidity can influence the availability of volatiles in the atmosphere by regulating their breakdown processes and the formation of secondary aerosols.^153,154^ In addition, a negative relationship between ozone levels and humidity is constantly observed in air quality data. High vapor pressure deficit (VPD) has a strong correlation with higher ozone levels.^155–157^ This correlation is partly caused by the lower dry deposition of ozone by plant stomata due to stomatal closure under high VPD.^158^ Since the late 1990s, there has been an increase in VPD consistently associated with the decrease in primary production, which can offset the positive effect of elevated CO_2_ on plant growth.^159^ This high VPD can affect the concentrations of ozone in the atmosphere and, therefore, also the chemical interactions between plant vegetative volatiles and ozone (see Section 4.1). The dynamics of these interactions, however, are still unknown and need further research especially in the context of plant ecological interactions.

In addition to atmospheric humidity, the humidity in the soil can influence the diffusion and degradation of belowground vegetative plant volatiles.^160^ In maize, for instance, the diffusion of β-caryophyllene produced by roots was found to be much faster in drier soils.^161^

### 4.3 Other organisms

Vegetative volatiles can be absorbed or degraded by other organisms after their release.^162^ In tomato, (*Z*)-3-hexenol from damaged neighboring plants can be taken up and converted into (*Z*)-3-hexenylvicianoside, which negatively influences herbivores.^163^ Uptake of plant volatiles by plants can also be observed at larger scales.^164^ In a winter wheat (*Triticum aestivum*) field, volatile emissions were observed to differ significantly across the growing season.^165^ Different types of vegetative volatiles exhibited specific bi-directional exchanges between the field and atmosphere, involving plants, soil, or both.^165^ Similar to the role of plants as volatile sinks, soil microbes can rapidly mineralize volatiles, a process that is highly dependent on the microbial community and specific volatile compounds.^166^

Vegetative volatiles can stick to the surface of other plants. This absorption can lead to reductions in atmospheric volatiles and re-emission of these volatiles into the atmosphere.^167–169^ Wild rosemary (*Rhododendron tomentosum*) is a strong volatile releaser that produces many insect-repelling volatiles. The volatiles released by *R. tomentosum* have been found to be adsorbed and re-release by neighboring birch and can lead to enhanced resistance of birch against green leaf weevils (*Polydrusus flavipes*).^169^ In summary, biotic interactions influence the availability of atmospheric vegetative volatiles. However, detailed knowledge on how this exchange of volatile between the biosphere and atmosphere is still lacking.

## 5. The role of vegetative plant volatiles in biotic interactions

**Fig. 4:**
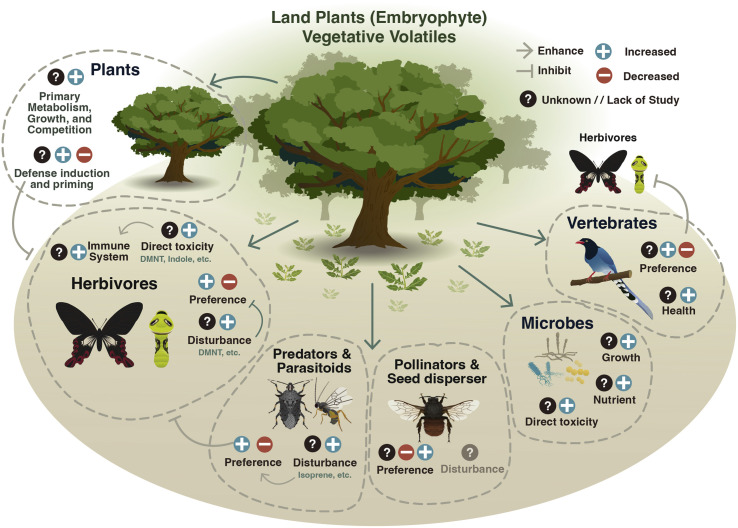
The role of vegetative plant volatiles in biotic interactions. Vegetative plant volatiles have important functions in interactions with other plants, invertebrates, vertebrates, and microorganisms. Vegetative plant volatiles can enhance (+), or suppress (−) biological and physiological functions in other organisms. In neighboring plants, vegetative plant volatiles can modulate defenses and growth. Invertebrate herbivores and pollinators can use vegetative plant volatiles to detect their preferred host or to avoid non-suitable ones. Antagonists of herbivores such as predators and parasitoids can also use vegetative plant volatiles as cues to locate herbivores. Vegetative plant volatiles can act as toxins against microorganisms or promote their growth. Vertebrates such as birds can also use volatiles to determine their foraging behavior. The diversity of biological effects of vegetative plant volatiles likely results in multifaceted ecological and evolutionary dynamics which shape the regulation of plant volatile production and release.

Once released, vegetative volatiles can influence the behavior and physiology of a wide range of organisms (Fig. 4). These effects have important consequences for ecosystem functioning and organismal behaviour.

### 5.1 Effects on plants

Vegetative plant volatiles reprogram plant defenses.^14,170^ Induced volatiles, for instance, act as stress cues that can be perceived by non-attacked tissues and neighboring plants and trigger defense responses.^170^ GLVs are released rapidly by plants upon wounding.^8,171,172^ GLVs such as hexenal, hexenol and hexenyl acetate can prime defenses in neighboring plants, whereby there is a potentiated defence capacity without induction until plants are subsequently challenged with a stressor.^8,171,173^ GLVs can also directly induce plant defense responses. In maize, exposure to (*Z*)-3-hexenol for 20–60 min induces defense gene expression to a much greater degree than other stress-induced volatiles (MeJA and MeSA).^174^ The sensitivity of plants to these early damage-induced volatile signals is likely to help them fend off subsequent attacks.^173^ Hexenal can be taken up by plants and enzymatically reduced into hexenol and subsequently into hexenyl acetate.^172,175^ Further, these GLVs are converted into defences (*e.g.*, GLV-glycosides) that can help plants combat environmental stressors.^176^

Indole is a nitrogen-containing volatile that is also emitted relatively early after attack.^32^ Indole can prime defense responses to herbivory or pathogen infection.^31,32,173,177^ In rice (*Oryza sativa*) and tea (*Camelia sinensis*) indole-mediated priming was shown to be dependent on mitogen-activated protein kinase and downstream WRKY transcription factors, suggesting that this priming occurs in a JA-dependent fashion.^31,178^ In maize, pre-treatment with indole primed up-regulation of anti-necrotrophic fungal defense genes and significantly reduced disease severity upon exposure to *Fusarium graminearum*.^177^ Indole also exhibits synergistic priming effects together with GLVs,^173^ demonstrating that different volatiles can act in concert to reprogram plant defenses.^179^

Other volatiles have also been shown to reprogram plant defenses^21^ and have been implicated in genotype-specific responses.^14,180,181^ Nonanal and β-ionone trigger systemic acquired resistance against the hemi-biotrophic pathogen *Blumeria graminis* in barley (*Hordeum vulgare*).^182^ Further, a blend of α- and β-pinene induced the production of reactive oxygen species and SA defences in Arabidopsis.^83^ The homoterpene DMNT induced the activity of Sporamin protease inhibitors, and when *Spodoptera* larvae were fed on DMNT-exposed plants, their performance was significantly reduced compared to unexposed plants.^183^ Exposure to plant volatiles can also alter plant primary metabolism. A recent study found that Scots pine seedlings exposed to vegetative volatiles released from large pine weevil (*Hylobius abietis*)-damaged plants display increased stomatal conductance and photosynthesis, enabling plants to accumulate a larger pool of C-based metabolites.^184^ Volatile information transfer between plants can also have negative consequences for receiver plants. For example, the blend of induced volatiles emitted from tomato plants following whitefly (*Bemisia tabaci*) infestation were shown to suppress anti-whitefly defences in receiver plants, rendering them more susceptible to whiteflies.^185^

In addition to inducible volatiles, constitutive volatile emission can also play a role in information transfer between plants. Volatiles constitutively emitted by spotted knapweed (*Centaurea stoebe*) roots were shown to increase carbohydrate and protein levels within the roots of common dandelion (*Taraxacum officinale*), which in turn made *T. officinale* plants more susceptible to cockchafer beetle (*Melolontha melolontha*) larvae.^186^ When potato (*Solanum tuberosum*) plants were exposed to vegetative volatiles constitutively emitted from onion (*Allium cepa*) plants, they emitted higher quantities of (*E*)-nerolidol and TMTT.^187^ Sesquiterpenes from the spotted knapweed such as β-caryophyllene, can promote growth and germination in sympatric neighboring plants, but the mechanisms of this interaction remain unclear.^188^

Plants are often grown in close proximity to other plants^24^ and are thus exposed to complex mixtures of constitutive and induced vegetative plant volatiles.^189,190^ The abundance and persistence of specific vegetative volatiles within plant canopies is dependent on the diversity and density of plants in a given ecosystem.^191^ However, we currently know little about the impact of volatile transfer across plant communities. Exposing healthy, uninfected *Arabidopsis thaliana* plants to monoterpenes emitted from plants infested with *Pseudomonas syringae* and then subsequently exposing the volatiles from a healthy plant to a third healthy plant enhances the resistance of the third plant to subsequent *P. syringae* infection.^192^ Thus, volatile defense cues may be transmitted across plant populations. Volatile blends of odor plumes become less similar to the original composition with increasing distance from the odor source, which may modulate population and community level effects.^193^ In beech (*Fagus*), volatile defense induction was strongest at distances <5–7 m, suggesting strong spatial dependency.^142^

### 5.2 Effects on invertebrates

#### 5.2.1 Herbivores

Herbivores respond behaviorally and physiologically to vegetative plant volatiles. They can use plant volatiles to locate host plants for feeding or oviposition.^194^ Hexenol, for instance, is attractive for the African cotton leafworm (*Spodoptera littoralis*) larvae.^195^ Indole can both attract or repel *S. littoralis* caterpillars depending on context and previous experience.^18,196^ The Western corn rootworm (*Diabrotica virgifera virgifera*) uses ambient plant-emitted CO_2_ for long distance host location, and volatiles such as (*E*)-β-caryophyllene for short range host location.^197–199^ Certain vegetative plant volatiles can also directly disrupt normal olfactory reception of plant-derived compounds by both herbivorous insects and their natural enemies. For example, DMNT, a compound which is typically emitted in very low amounts in the absence of stress, and is highly induced during herbivory, has been shown to suppress the activity of herbivore olfactory neurons tuned to host plant attractant chemicals, limiting the capacity for herbivores to effectively locate plants.^200,201^

Volatiles can also act as repellents against herbivores in a temporally dependent fashion. In tobacco, herbivore-induced volatiles emitted at night repel nocturnal ovipositing by the tobacco budworm (*Heliothis virescens*) moths.^202^ Furthermore, some volatiles can act directly as toxins against herbivores. DMNT can damage the peritrophic matrix in the diamondback moth (*Plutella xylostella*) midguts making them more susceptible to microbial infection and, ultimately, leading to strong reductions in herbivore performance and survivability.^203^ Indole has also been shown to reduce the survival of the beet armyworm (*Spodoptera exigua*) when infused into artificial diet.^204^ Exposure to natural blends of tomato volatiles is associated with increased expression of cytochrome p450 enzymes and increased larval growth on artificial diet containing trypsin inhibitors, suggesting that plant volatiles may boost herbivore detoxification processes.^205^

Volatile concentration plays a crucial role in determining their effects on herbivores. For example, attraction to indole of *S. littoralis* caterpillars is strongest at lower concentrations whereas attraction to hexenol is strongest at higher concentrations.^195^ Indole can also repel *S. littoralis*, an effect that is lost when the caterpillars are in the proximity of parasitoids.^18^ The performance of the Western corn root worm was shown to be dependent on the density of conspecific larvae also feeding on maize roots, further, larvae used (*E*)-β-caryophyllene emission levels associated with the suitable density of conspecifics to locate host plants.^206^ Additionally, in cabbage, only certain herbivore species resulted in dose-dependent volatile emission. When fed upon by cabbage white (*Pieris rapae*) plants emitted volatiles in a larval-density-dependent fashion with commensurate parasitoid attraction. However, when fed upon by the *P. xylostella*, volatile emission was constant across varying larval densities.^207^

#### 5.2.2 Predators and parasitoids

Predators and parasitoids use induced vegetative volatiles as host locating cues (reviewed in detail by ref. 7). GLVs in specific isomeric ratios can attract *Geocoris* spp. predatory bugs to *Nicotiana attenuata* plants.^76^ In black poplar (*Populus nigra*), the braconid parasitoid *Glyptapantales lipardis* is attracted to 2- and 3-methyl butyraldoxime. These compounds are only released from damaged tissues and not systemically, which could provide natural enemies with the precise feeding location of herbivores. Although damaged sites typically emit higher levels of volatiles, many volatiles are released from systemic tissues during herbivore feeding.^15^ Induced volatile emission can also directly modify the attractiveness of herbivores to natural enemies. For example, the predatory mite *Phytoseiulus persimilis* was more attracted to volatile blends released by spider mite-infested plants compared to *Spodoptera exigua*-infested plants.^208^ Exposure to indole modified the overall scent of *S. littoralis* larvae, rendering them less attractive to the endoparasitoid *Miroplitis rufiventris*. Interestingly, although indole acts as a repellent for *Spodoptera littoralis* in the absence of *M. rufiventris*, when parasitoids are present the repellence effect is diminished.^18^ Complex ecological interactions can modify volatile-mediated host location by herbivore natural enemies. *Brassica nigra* plants exposed to root feeding by cabbage root fly (*Delia radicum*) larvae and leaf feeding by the large white (*Pieris brassicae*) larvae are more attractive to the parasitoid *Cotesia glomerata* than plants infested with *P. brassicae* larvae alone.^209^ Parasitism by *C. glomerata* alters the composition of *P. rapae* larval oral secretions and modulates herbivore-induced plant volatile blends, which attracts hyperparasitoids.^210^ Finally, isoprene, whose emission is mainly regulated by abiotic stress, can interfere with the attraction of the parasitic wasp *Diadegma semiclausum* to Arabidopsis.^211^ These studies highlight how the diversity of volatiles and their regulation leads to complex outcomes in multitrophic interaction networks.

#### 5.2.3 Pollinators and seed dispersers

The behavior of pollinators and seed dispersers is often determined by vegetative plant volatiles.^4^ Interactions between stress-induced volatile emission and pollinators can yield complex ecological outcomes. When infected by *Cucumber mosaic virus* (CMV), resultant changes in volatile emission from tomato plants increased attractiveness of plants to pollinating bumble bees (*Bombus terrestris*). This increase in attraction further led to enhanced pollination of infected tomato flowers, which counteracted the negative impact of CMV infection on seed production.^91^ Volatiles can also have multiple functions for a single herbivore species. Although stress-induced volatiles have been shown to deter foliar oviposition by tobacco hornworm (*Manduca sexta*) adults on tobacco plants, the same volatiles were also shown to attract foraging *M. sexta* adults to flowers and thus enhance pollination.^212^ In agarwood (*Aquilaria sinensis*), GLVs released from seeds can attract *Vespa* hornets, which are important seed dispersers. Additionally, many of these volatiles are similar to those induced during foliar herbivory; this has been suggested to be a repurposing of plant defense to facilitate effective seed dispersal.^213^ Herbivore-induced vegetative volatile emissions can also have negative impacts on plant–pollinator relationships. Vegetative plant volatiles released from wild tomato (*Solanum peruvianum*) plants under both real and simulated (MeJA application) herbivory conditions reduced the plant attractiveness to pollinators. This resulted in a potential decrease in fitness due to reduced seed set.^214^

### 5.3 Effects on vertebrates, including humans

Vegetative plant volatiles can influence the behavior and performance of larger vertebrates. The great tit (*Parus major*) can distinguish between herbivore-infested and herbivore-free apple trees using volatile cues.^215^ Terpenes such as α-farnesene and DMNT have been implicated as potential cues as both are significantly induced in herbivore-infested plants, which are more attractive to insect-eating birds.^215–217^

Many reptiles are also olfactory foragers. The active foraging lizard (*Sceloporous virgatus*) responds to the GLV (*E*)-2-hexenal primarily by inducing tongue-flicking, which is a known chemosensory behavior.^218^ Apart from this example, little is known about the impact of plant volatiles on reptiles.^218,219^

Several herbivorous mammals are known to use vegetative plant volatiles as foraging cues. Goats respond negatively to terpenes emitted by galls formed during gregarious aphid (*Salvum wertheimae*) infestation of wild pistachio (*Pistacia atlantica*) trees.^220^ Elephants use the monoterpenes β-ocimene and linalool, the sesquiterpenes β-caryophyllene and 1-methylpyrrole, as well as the tryptophan-derived indole for food choice.^221^

Vegetative plant volatiles can also directly impact human behaviour. Certain volatile compounds in edible foods such as coriander (*Coriandrum sativum*) have been linked with positive emotions, increased salivation, and enhanced theta band activity in the cerebral cortex.^222^ Additionally, several studies have highlighted the positive impacts of plant-dominated areas on mental health.^223,224^ Forest bathing, an activity whereby a person spends a prolonged period in a forest, is associated with the amelioration of a number of physical ailments and has been partially linked to volatile cues.^225^*In vitro*, 1,8-cineole and β-caryophyllene have been shown to reduce neuro-inflammation (associated with disorders such as Alzheimer's disease).^226,227^ Phellandrene, a terpene emitted by a wide range in plants has also been associated with anti-inflammatory response in human cell cultures.^228^ There is evidence that inhaled terpenes such as limonene and pinene can end up in the human bloodstream, although considering they are expelled in unchanged form, they do not seem to be metabolized.^225,229^

### 5.4. Effects on microorganisms

Vegetative plant volatiles influence interactions of plants with multitude of microorganisms, such as viruses, bacteria, archaea, protozoa, fungi, algae, and nematodes.^230–232^ Plant-derived terpenoids, benzenoids, and aldehydes can all exert antibacterial or antifungal effects. For instance, the monoterpenes limonene and β-pinene, and several aldehydes, which are major constituents of the volatile bouquet emitted by pine trees, display antimicrobial activities against airborne bacteria.^233^ Similarly, GLVs such as (*E*)-2- and (*Z*)-3-hexenal show antimicrobial effects against plant pathogenic bacteria *in vitro*.^234^ Yet, exceptions to this pattern have been reported for the sesquiterpene (*E*)-β-caryophyllene, which can directly stimulate the growth of the hemi-biotrophic fungus *Colletotrichum graminicola*.^235^ In rice, the induced emission of the monoterpene (S)-limonene upon infection with the pathogenic fungus *Magnaporthe oryzae* can inhibit the germination of *M. oryzae* spores.^236^

Besides antimicrobial activities, vegetative plant volatiles can influence the establishment of microbial communities by acting as attractants, nutrients, or substrates to produce bioactive compounds.^237^ Linalool, 2-phenylethanol, and nonanal emitted by strawberry (*Fragaria ananassa*) plants can be chemically transformed by leaf epiphytic bacteria and enhance their competitive performance in the presence of the pathogenic fungi *Botrytis cinerea*.^238^ Root-derived volatiles emitted by tomato plants can attract certain soil bacteria.^239^ These authors also observed that some bacteria might be more strongly attracted by plant volatiles under soil nutrient limitation, implicating root volatiles as info-chemicals that provide information about a nearby nutrient-rich environment. Whether active recruitment of microorganisms by vegetative plant volatiles affects plant performance or resilience against biotic and abiotic stressors, however, is not well explored.

In addition to direct effects, microbial communities can indirectly be affected by the interplay of vegetative plant volatiles with other plants or higher trophic organisms. For instance, pathogen-induced emission of aboveground vegetative volatiles in infected plants can alter the responses of non-infected neighbors to the same microbial pathogen either by increasing or reducing susceptibility.^240^ More recently, vegetative volatiles emitted aboveground by tomato plants inoculated with beneficial root-associated bacteria were shown to modulate the rhizosphere microbiota of surrounding conspecific plants *via* volatile-induced changes in root exudates.^241^ The ecological and physiological implications for both the emitter and receiver plants, as well as their associated soil microbiota, were not further investigated. Still, this novel phenomenon adds further support for the potential use of above- and/or below-ground volatiles to fine-tune the rhizosphere microbiota in agricultural systems.

## 6. Synthesis: biological frameworks to explain patterns of vegetative plant volatile release

**Fig. 5:**
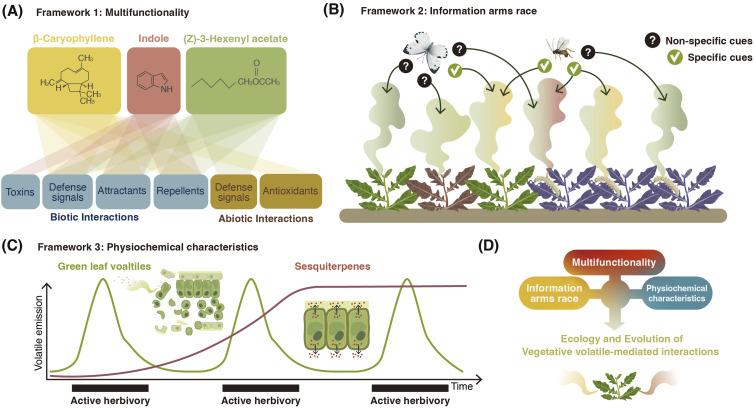
Biological frameworks that help to explain release patterns of vegetative plant volatiles. (A) Multifunctionality: plant volatiles can serve multiple purposes for plants, and this multifunctionality is mirrored in the regulation of their production and release. (B) Vegetative volatiles provide information to both allies and antagonists, which may result in an information arms race that shapes the composition of plant volatile bouquets. Different colors depict different blends of volatiles, some of which contain useful information only for beneficials and others can act as useful signals for both beneficials and enemies. (C) Physiochemical characteristics such as the biochemical origin, the volatility, the stability and the toxicity of vegetative volatiles influence their production and release kinetics, which again determines potential functions and environmental effects. (D) Interactions between A, B, and C will influence the evolution of vegetative plant volatiles, and thus help to explain spatiotemporal production and release patterns that are observed in nature.

Vegetative plant volatiles are produced *via* a multitude of pathways and follow different routes to end up in the environment. The biosynthesis and release of volatiles is modulated by both biotic and abiotic factors. Together, this results in highly dynamic and diverse volatile release patterns. Once the volatiles have left the plant, they interact with organisms of all known kingdoms, both at behavioral and physiological levels. These interactions again shape the genetics of volatile release and production over evolutionary time. Given this substantial physiological and ecological complexity, one has to wonder whether there are any specific biological frameworks beyond the most fundamental physical, chemical, and biological principles that explain vegetative volatile release patterns. Over the last years, a number of frameworks have started emerging that may help to explain volatile release patterns (Fig. 5).

### 6.1. Multifunctionality

There is now ample evidence that the same volatile can have multiple functions for plants^242^ (Fig. 5). GLVs, for instance, act as within-plant defense signals, antimicrobials, and attractants of natural enemies.^8^ Indole primes plant defenses, it acts as a repellent at high doses, and it can directly intoxicate herbivores.^18,32,196,204^ The homoterpene DMNT can act as an herbivore toxin when ingested, and it also interferes with the location of the herbivore's host plant when perceived by olfactory receptors.^201,203^ β-caryophyllene has been implicated in biotic interactions as a volatile cue both above- and below-ground, where the impacts on plants range from beneficial to detrimental. For example, β-caryophyllene can attract herbivores' natural enemies^243^ or help *Diabrotica virgifera virgifera* larvae to locate their host plant roots.^206^ β-caryophyllene has also been implicated as a critical defense against the fungal pathogen *Pseudomonas syringae*.^244^ The regulation of the production and release of these compounds likely mirrors their multiple roles and benefits for plants. During insect herbivory, biosynthetic similarity between compounds is associated with higher covariance between compounds, suggesting that there may be constraints at the biosynthetic level for covariation patterns between different volatile groups.^245^ For example, multiple compounds can be produced from a single enzyme/substrate pair. Further, conditions such as catalytic metal ion availability and pH can influence the relative abundances of terpene products *in vitro*.^246^ It is thus possible that, under variable conditions within plant tissues, the relative abundance of products of the same substrate/enzyme might vary. Developing a deeper understanding of the multifunctionality of volatiles can thus help to uncover ecological and evolutionary rationales for their biosynthesis and emission.

### 6.2. Chemical communication displays and the information arms race

Vegetative plant volatiles can provide information to beneficial organisms such as herbivore natural enemies, but also to antagonists such as herbivores. These conflicting functions and effects can be described as an information arms race, whereby the plant tries to hide from herbivores while being visible to herbivore natural enemies.^247^ In this framework, the ability of plants to produce volatiles that are protective but not necessarily unique (*i.e.*, other nearby plants are also producing them) is most effective for minimizing fitness costs associated with volatile emission.^247^ Volatile emission can be considered as a chemical communication display that serves as information (Fig. 5B). In plants, covariation between volatiles is relatively low, particularly following herbivory, suggesting that single or few compounds are likely responsible for mediating a particular information transfer between a plant and another organism.^245^ If herbivores rely on fixed ratios of compounds, then the further reduction of covariance among herbivory-induced plant volatiles may interfere with the host location of subsequent herbivores.^248^ Further exploration regarding how multifunctionality, not only of the volatile pool but also of individual compounds, influences the ecological interactions in complex ecosystems is essential to identify how volatiles ultimately impact plant fitness and evolutionary trajectories.

### 6.3. Physiochemistry

Depending on their physiochemical characteristics, vegetative plant volatiles show distinct spatiotemporal release and transfer patterns. Some are breakdown products and thus released rapidly from wounded tissues, while others are synthesized *via* enzymatic cascades and thus produced more slowly^8,32^ (Fig. 5C). Some are stable and diffuse across large distances, and others are unstable and rapidly degraded.^249^ Thus, vegetative plant volatiles have distinct physiochemical fingerprints, which constrain their adaptive plasticity and ecological effects. GLVs, for instance, are breakdown products rapidly released following stress, mostly from wound sites.^250^ They thus provide a timely, but unspecific cue of active damage, and may be used by natural enemies accordingly.^251^ Similarly, the above properties predispose GLVs to act as damage-associated molecular patterns in plant defense regulation.^252^ Volatiles such as terpenes, on the other hand, diffuse slowly and take longer periods to be produced because of dedicated biosynthetic machinery. Accordingly, they can act as highly specific cues for certain herbivores and other plants, as well as constitutive defenses or phytoanticipins. Hence, terpenoids might be less suited as rapid, localized defense signals (Fig. 5C). There are, however, exceptions to this; terpenes stored in trichomes for instance can be released rapidly upon damage (see Section 2.2).

In summary, we propose that the release patterns of a given class of volatiles can be explained, at least in part, by their multifunctionality, the information arms race, and their physiochemical properties. A better understanding of the physical properties and ecology of individual volatile classes is indispensable to leverage the power of these frameworks (Fig. 5D).

## 7. Open questions and future research on vegetative plant volatiles

Although the field is rapidly gaining a deep understanding of the physiology and ecology of vegetative plant volatiles, several important open questions remain.

### 7.1 Mechanisms of transport and release

While the biosynthesis of vegetative plant volatiles is understood in substantial detail, the mechanisms governing transport and release are not well known yet (Fig. 2). Most molecular work so far has focused on floral volatiles,^253^ and it remains to be seen whether vegetative volatile release is governed by the same mechanisms. A particular challenge will be to understand the relative importance of transport and/or diffusion across cell membranes, cuticular barriers, stomata, trichomes, and wounds. Given the vast diversity of physiochemical characteristics across vegetative plant volatiles, it is likely that different volatiles will have different preferred release pathways mediated by the physiological characteristics of plant tissues.^41,44^ Understanding the major transport routes of individual volatiles will be imperative to investigate their regulation, ecology, and evolution in an environmental context.

### 7.2 Interactions of multiple stresses on volatile production and release

Plant volatile responses to individual biotic and abiotic factors are well described, and we have also gained more detailed understanding on how different abiotic factors interact in this context.^254^ However, much remains to be learned regarding the impact of multivariate environments on plant volatile production and release. Interactions between multiple abiotic factors at different levels of stress severity, for instance, are not well known. Severe stress might trigger completely different responses in comparison to mild stress. Climate change has led to an increase in multi-stress scenarios worldwide,^114^ which will most likely affect plant volatile emissions and plant interactions as well. Investigating how plant volatiles profiles respond to these dynamic abiotic pressures will help to predict plant performance in the context of herbivory and beyond.

### 7.3 Transmission dynamics in the field

A number of methods has been developed to detect, identify, and quantify plant volatiles.^255^ The pros and cons of various plant volatile measurement techniques are reviewed in detail by Tholl *et al.*^256^ Most of these methods rely on collection approaches with low temporal resolution and/or involve highly restricted headspace environments such as glass bottles.^257,258^ For example, volatiles are often collected and concentrated over time (often several hours) and collections are then analyzed using techniques such as gas chromatography coupled with mass-spectrometry (GC-MS).^256^ As samples are pooled, the precise time point in which these volatiles are emitted is difficult to determine. Even if samples are collected for shorter periods of time, materials and lengthy analytical program run times can cause temporal experimental bottlenecks. Techniques such as proton-transfer reaction time-of-flight mass spectrometry (PTR-ToF-MS) do not require a time-consuming separation phase and allows for direct injection of airborne volatiles into the mass spectrometer. Still, PTR-ToF-MS cannot differentiate chemical structures with identical molecular weights, *e.g.*, different monoterpenes.

It is clear that there is high spatial variability in vegetative plant volatile emissions, whether it be differing plant tissues and plant species or between plants under variable environmental pressures.^259–261^ Capturing this variation in more detail will be important in the future. Recently, technological advancements have allowed for real-time measurements of volatiles across multiple plants, however this has yet to be implemented in a field setting.^130^ By consequence, we lack a detailed understanding of spatial and temporal volatile dynamics at the plant and interplant scale within plant canopies.^256^ Developing our understanding of how vegetation creates a “volatile landscape” in terrestrial ecosystems is important to understand how ecosystem-scale volatile dynamics mediate ecosystem functioning. The development of adequate devices/technologies will play an essential role in the study of plant volatiles at the canopy level.

### 7.4 Mechanisms of uptake, perception, and physiological responses

Plant volatiles are perceived and, in some cases, taken up by plants and other organisms.^163,179,262–265^ The mechanisms underlying these processes, however, are not well understood. Volatile uptake by plants, for instance, is thought to occur through the stomata or *via* diffusion through the leaf cuticle,^253,266^ but neither of these mechanisms have been explored in detail. To date, a receptor-based mechanism in plants has only been reported for the volatile phytohormone ethylene.^267^ Volatile perception has been explored at the molecular level for a number of insects, and different mechanisms are being validated for plants.^164,268,269^ Outside of perception, volatiles' targets that may trigger physiological responses in insects and mammals are unknown and deserve further study to harness the potential of plant volatiles as pest control agents and drugs.

### 7.5 Identifying major evolutionary forces driving vegetative plant volatile patterns

Given the many functions and effects of vegetative plant volatiles, understanding the major forces that govern their evolution remains challenging. Volatiles have been implicated as driving forces in shaping resistance to herbivory in receiver plants, sometimes in a taxa specific manner. There is also genotype-specific information transfer between plants, where signals from kin have been shown to result in the highest level of resistance to herbivory in receiver plants.^14,270^ The existence of elicitors and effectors produced by both arthropods and microbes modulating volatile emissions has reinforced the idea that vegetative plant volatiles are important actors in the ecology of plant biotic interactions and might have shaped the evolution of these traits.^43,271^ In nature, however, plants are exposed to all these components either simultaneously or sequentially through their lifetime. Thus, it is likely that volatile-related traits have evolved under the influence of a dynamic environment. Understanding how vegetative plant volatiles respond to multiple biotic factors is, therefore, essential to unravel their ecological functions.

## 8. Author contributions

All authors conceived the idea for this review. REB, PAL and JMW contributed equally to the writing of the first draft (order of authorship determined by alphabetical order of last name). ME wrote additional sections and made critical revisions to subsequent drafts. All authors made substantial contributions to the final draft.

## 9. Conflicts of interest

The authors have no conflicts of interest to declare.

## Supplementary Material
